# Artichoke Polyphenols Sensitize Human Breast Cancer Cells to Chemotherapeutic Drugs via a ROS-Mediated Downregulation of Flap Endonuclease 1

**DOI:** 10.1155/2020/7965435

**Published:** 2020-01-03

**Authors:** Anna Maria Mileo, Donato Di Venere, Stefania Mardente, Stefania Miccadei

**Affiliations:** ^1^Tumor Immunology and Immunotherapy Unit, IRCCS Regina Elena National Cancer Institute, Rome, Italy; ^2^CNR-Institute of Sciences of Food Production (ISPA), Bari, Italy; ^3^Department of Experimental Medicine, Sapienza University of Rome, Rome, Italy

## Abstract

Combined treatment of several natural polyphenols and chemotherapeutic agents is more effective comparing to the drug alone in inhibiting cancer cell growth. Polyphenolic artichoke extracts (AEs) have been shown to have anticancer properties by triggering apoptosis or reactive oxygen species- (ROS-) mediated senescence when used at high or low doses, respectively. Our aim was to explore the chemosensitizing potential of AEs in order to enhance the efficacy of conventional chemotherapy in breast cancer cells. We employed breast cancer cell lines to assess the potential synergistic effect of a combined treatment of AEs/paclitaxel (PTX) or AEs/adriamycin (ADR) and to determine the underlying mechanisms correlated to this potential therapeutic approach. Our data shows that AEs/PTX reduced cell proliferation by increasing DNA damage response (DDR) mediated by Flap endonuclease 1 (FEN1) downregulation that results into enhanced breast cancer cell sensitivity to chemotherapeutic drugs. We demonstrated that ROS/Nrf2 and p-ERK pathways are two molecular mechanisms involved in the synergistic effect of AEs plus PTX treatment. To highlight the role of ROS herein, we report that the addition of antioxidant N-acetylcysteine (NAC) significantly decreased the antiproliferative effect of the combined treatment. A combined therapy could be able to reduce the dose of chemotherapeutic drugs, minimizing toxicity and side effects. Our results suggest the use of artichoke polyphenols as ROS-mediated sensitizers of chemotherapy paving the way for innovative and promising natural compound-based therapeutic strategies in oncology.

## 1. Introduction

Breast cancer is the most common malignancy in women around the world [1] and is a heterogeneous disease with high degree of diversity between and within tumors and among individual patients [2–4]. Of the various factors involved in breast carcinogenesis, oestrogen receptors (ER) play a major role and are considered an important therapeutic target. ER-positive tumors are further subtyped into low proliferation rate luminal A and higher proliferation rate luminal B tumors. Patients with the triple negative breast cancer (TNBC) subtype, characterized by the absence of ER, progesterone receptor (PR), and human epidermal growth factor receptor-2/neu receptors (HER2/neu) have a poor prognosis [5, 6] also due to the few clinical treatments available. Considerable effort has gone into identifying new therapeutic agents, with multiple targeting abilities, able to circumvent the limitation of current conventional therapy.

Combined cancer therapy utilizes two or more agents and may improve the therapeutic efficacy of the single drug through a synergistic effect, leading to a potentially reduced drug resistance [7].

Many epidemiological studies suggest that phytochemicals, present at high levels in vegetables and fruits, have anticarcinogenic properties [8–11] and, triggering apoptosis, may be an effective treatment in cancer.

There is considerable interest in identifying bioactive compounds which, by increasing the sensitivity to conventional chemotherapeutic agents, could improve the patient's quality of life by reducing the side effects of therapy [12–17]. It has been recently demonstrated that combined treatment of natural polyphenols and chemotherapeutic agents are more effective than the drug alone in hindering the growth of cancer cells [18, 19] and in promoting chemosensitivity in multidrug resistance (MDR) cancer cell lines [20].

Growing interest in dietary phytochemicals has led to renewed attention being paid to the artichoke, because of its high content in polyphenols. Artichoke polyphenols are mainly glycoside forms of flavonoid, such as apigenin and luteolin in the leaves and hydroxycinnamic acid derivatives in the edible part, mainly represented by mono- and dicaffeoylquinic acids. Many *in vitro* and *in vivo* experiments have shown that artichoke has diuretic, hepatoprotective, hypocholesterolemic, and antioxidant properties [21–24] and, more recently, antitumoral activities [24–26]. Our previous findings indicate that AEs protect hepatocytes from oxidative stress and show cancer chemopreventive properties by triggering apoptosis in human hepatoma cells [24] and in human breast cancer cell lines without any toxicity in the nontumorigenic MCF10A cells [25]. We have also provided evidence that low doses and chronic AE treatments exert anticancer activity through induction of premature senescence in MDA-MB231, a triple negative and highly aggressive breast cancer cell line [27]. Furthermore, the bioavailability of metabolites of hydroxycinnamic acids, after ingestion of cooked artichoke, has also been demonstrated in human subjects [28].

Taxanes are a family of chemotherapeutic drugs employed for the treatment of many tumors including breast cancer in both early and metastatic stages [29]. One of these, PTX, is a microtubule-stabilizing drug [30] which, because of its effect on mitotic spindle dynamics, may lead to cell cycle arrest and apoptosis [31]. More recently, it has been suggested that many anticancer drugs, including taxanes, have the ability to induce oxidative stress [32], which indicates an additional antitumoral mechanism.

FEN1 is a key member of the endonuclease family involved in cellular DNA replication and repair [33]. As a structure-specific nuclease, FEN1 stimulates Okazaki fragment maturation during DNA repair and efficient removal of 5′-flaps during long-patch base excision repair [34]. FEN1 is also reported to be linked to apoptosis-induced DNA fragmentation in response to apoptotic stimuli [34, 35], and its expression is closely associated with cell proliferation and correlated with increased tumor grade and aggressiveness [36].

Oxidative stress is a result of a cellular imbalance in the production of ROS and the activity of the endogenous antioxidant protective system [37]. Notably, ROS-induced oxidative stress plays an important role in cancer development and progression. Among ROS-inducing agents, many phytochemicals, including curcumin [38, 39], resveratrol [40, 41], and epigallocatechin-3-gallate [42], have been shown to enhance the anticancer properties of chemotherapeutic agents. We have previously demonstrated a dual role of AEs, as prooxidant in breast cancer cells [25] and as antioxidant in normal hepatocyte [24] showing an inhibitory effect on growth of tumor cells with little or no toxicity on normal cells based on their differential redox status.

It is well known that induction of phase II enzymes, counteracting reactive electrophiles including ROS, plays an important role in response to many anticancer agents including dietary compounds. Upon cellular stimulation by oxidative stressor molecules Nrf2, the main transcription factor involved in the regulation of phase II and antioxidant gene expression moves to the nucleus where it interacts with antioxidant response elements (AREs) present in the promoter region of many phase 2 genes [43–45].

The roles of growth factors and mitogens in regulating gene expression, apoptosis, and differentiation have been reported to be mediated by the Ras/MEK/ERK signaling cascade [46]. This pathway has been shown to be frequently activated in breast cancer, and the MAPK pathway is a well-explored target of therapeutic intervention. Therefore, specific inhibitors targeting Ras, Raf, MEK, and other downstream proteins have been tested in clinical trials [47]. Since p-ERK expression sensitizes activation of DNA damage-induced checkpoints, inhibition of p-ERK may enhance the genotoxic effect of chemotherapy, probably as a consequence of the accumulation of DNA lesions due to compromised checkpoint activation [48].

To improve our knowledge for designing new therapeutic interventions, we have characterized the biological role of the combined treatment involved in the synergistic antitumor effect of AE/PTX approach.

## 2. Materials and Methods

### 2.1. Artichoke Extract Preparation

The edible part (head) of fresh artichoke is used for extract preparation, and the analysis of polyphenols contained in the extracts was performed by HPLC as previously described [25].

### 2.2. Cell Lines and Cultured Conditions

The human breast cell lines were maintained in a humidified incubator with 5% CO_2_ and 95% air at 37°C. MCF7 and MDA-MB231 cells, respectively, luminal A oestrogen receptor positive and basal B triple negative receptor subtypes were grown in RPMI and DMEM, respectively (Invitrogen, Life Technologies, Monza, Italy) and supplemented with10% FBS, 10 IU/ml of penicillin and 10 *μ*g/ml of streptomycin.

### 2.3. Reagents

Artichoke extracts were dissolved in phosphate buffer solution (PBS) and 0.1% Me_2_SO (Sigma-Aldrich, Milan, Italy). Chemotherapeutic drugs PTX (Sigma-Aldrich), ADR (Sigma-Aldrich), and cisplatin (CDDP; Sigma-Aldrich) were dissolved in PBS. Glucose oxidase (GOx, Sigma-Aldrich) was dissolved in PBS. ERK1-2 inhibitor 1,4-diamino-2,3-dicyano-1,4-bis(2-aminophenylthio) butadiene (U0126, Promega, Milan, Italy) was dissolved in Me_2_SO. NAC (Sigma-Aldrich) was dissolved in PBS. Dihydroethidium (DHE) and dichlorofluorescein-diacetate (DCF-DA, Molecular Probes-Thermo Fisher Scientific, Waltham, MA, USA) were dissolved in Me_2_SO.

### 2.4. Cell Viability Assay

Cells were seeded in 96-well plates at a concentration of 3 × 10^3^ cells/well and after 24 h treated with drugs at the given concentrations. CellTiter-Glo Luminescent Cell Viability Assay (Promega) was used to determine the relative number of viable cells after treatment, by means of a GLOMAX 96 Microplate Luminometer (Promega). Cells treated with the same final concentration of drug solvent were used as control.

### 2.5. Colony-Forming Assay

MCF7 cells were plated at a concentration of 3.5 × 10^3^ cells/well in 6-well plates. After 24 h, vehicle, PTX, AEs, or a combination of both (as indicated) were added for 24 h. After 14 days, cells were washed and subsequently stained using a 5% crystal violet solution in order to assess the colony number.

### 2.6. Immunoblot Analysis

To obtain the whole-cell extract, cells were washed with PBS and suspended in RIPA lysis buffer in the presence of protease and phosphatase inhibitors. After 30 minutes in ice, samples were sonicated and centrifuged (10,000x*g*) for 10 min at 4°C. Supernatants were collected as whole-cell extracts. To obtain nuclear proteins, cell pellets were swelled in hypotonic buffer (Tris-HCl pH 7.5 50 mM, NaCl 10 mM, EDTA 5 mM, NP40 0.05%) for 30 minutes in ice. The cell lysate was centrifuged at 14,000 rpm × 30 minutes at 4°C. The supernatant was collected as the cytoplasmic fraction. The pellet was resuspended in buffer C (Hepes pH 7.9 20 mM, NaCl 420 mM, MgCl_2_ 1.5 mM, EDTA 0.2 mM, glycerol 25%, and protease inhibitors) and sonicated. Cellular debris was removed by centrifugation at 13,000 rpm at 4°C for 30 minutes. The protein content was determined with a protein assay reagent (Bio-Rad, Milan, Italy), using bovine serum albumin as a standard. An equal protein content of total cell lysates was resolved on polyacrylamide gel (Bolt 4-12% Bis-Tris Plus, Invitrogen) with molecular weight markers (BenchMark Pre-Stained Protein Standard, Life Technologies, Monza, Italy). Proteins were then electrotransferred to PVDF membrane (iBlot Invitrogen) and incubated with specific primary antibodies. Antibodies used for western blots were anticleaved PARP (Cell Signaling Technology, Danvers, MA, USA, # 9541 dil. 1 : 1000), anti-LC3 (MBL International, Woburn, MA, USA, PD014 dil.1 : 400), anti-ERK1-2 (Cell Signaling Technology #9102 dil.1 : 1000), anti-pERK1-2 (Cell Signaling Technology # 9101S dil.1 : 1000), anti-FEN1 (Santa Cruz Biotechnology Inc. Dallas, TX, USA, sc-28355 dil.1 : 1000), anti-*β*-actin (MP # 69100 dil.1 : 10000), anti-GAPDH (Sigma Aldrich G8795 dil. 1 : 24000); anti-Nrf2 (Cell Signaling Technology #8882 dil.1 : 1000), anti-gamma-H_2_AX (phospho S139) (Millipore # 05363 dil. 1 : 500), anti-histone H3 (Abcam, Milan, Italy, ab1791 dil.1 : 1000). PVDF membranes were developed using ECL detection reagents (GE Healthcare, Marlborough, MA, USA) on a UVITEC imaging system (UVITEC Cambridge, UK). Western blot signals were quantified by densitometry analysis using ImageJ software.

### 2.7. [5′-^3^H] Thymidine Incorporation Assay

Cells were seeded in 6-well plates at a concentration of 1.5 × 10^5^. After 24 h, vehicle, PTX, AEs, or a combination of both (as indicated) was added and the culture was incubated for 24 h. 37 kBq of [5′-^3^H] thymidine (DuPont, New England Nuclear Research Products, Boston, MA, USA) was added to each well. Four hours later, cells were washed twice with ice-cold PBS and 10% trichloroacetic acid (TCA). Cells were lysed in the presence of 1 N NaOH-0.1% SDS and neutralized in 1 N HCl. The cell-associated radioactivity was determined by liquid scintillation counting (Tri-Carb 2800 TR, PerkinElmer, USA).

### 2.8. RNA Extraction, Reverse Transcription, and Quantitative RT-PCR

Total RNA was extracted from MDA-MB231 cells using the MasterPure RNA Purification Kit (Epicentre Biotechnologies, Madison, WI, USA). RNA was reverse-transcribed into cDNA using the High Capacity cDNA Reverse Transcription Kit (Applied Biosystems Inc., Foster City, CA, USA) and subject to StepOne Real-Time PCR (Applied Biosystems Inc.) with PowerUp SYBR Green Master Mix (Applied Biosystems Inc.). Primers for FEN1, Nrf2, and GAPDH were designed as specified below:

FEN1 forward 5′-GCCAAAAAGCTGCCAATCCA-3′, FEN1 reverse 5′-GCCAATTTTCTGGCACAGGG-3′; Nrf2 forward 5′-CATCGAGAGCCCAGTCTTC-3′, Nrf2 reverse 5′-CTTCTGGACTTGGAACCATG-3′; and GAPDH forward 5′-TCCCTGAGCTGAACGGGAAG-3′, GAPDH reverse 5′-GGAGGAGTGGGTGTCGCTGT-3′.

PCR conditions were 50°C for 2 min, 95°C for 2 min, followed by 40 cycles of 95°C/15 s, annealing at 56°C/30 s and 72°C/30 s. All reactions were performed in triplicate. Data was normalized to GAPDH and the fold change in gene expression relative to normal was calculated using the comparative *Ct* method [49].

### 2.9. ROS Detection

#### 2.9.1. Fluorescence Microscopy

MDA-MB231 cells were treated with vehicle or PTX plus and minus AEs and GOx as positive control (as indicated). After 4 h, the oxidation-sensitive fluorescent probe DHE was used to assess the production of cytosolic superoxide anions. Briefly, after exposure, cells were incubated with 5 *μ*M DHE for 40 minutes at 37°C in the dark and then rinsed twice with PBS. The cell-permeant DHE entered the cells, was oxidized by superoxide anions to form ethidium (ETH) which binds to DNA, and produced fluorescent ETH-DNA. The fluorescent signals were obtained by the cultured cells at *λ*_ex_ 300 nm and *λ*_em_ 610 nm. Cells were visualized and images were captured using a fluorescence microscope apparatus (Olympus IX71-Olympus Tokyo, Japan) equipped with a digital camera (Tucsen Photonics Co., Ltd., Fuzhou, Fujian, China).

#### 2.9.2. Flow Cytometry Assay

ROS formation in MDA-MB231 cells with PTX and AEs was assayed by flow cytometry with the dye DCF-DA and following standard methods [50]. Briefly, DCF-DA (final concentration 40 *μ*M) was added to cell cultures on 6-well plates for 15 min at 37°C. After incubation, cells were scraped, washed in PBS, and analyzed by a flow cytometer (Epics XL-MCL Coulter, CA, USA) with an argon laser at 488 nm. Cells were gated using forward angle light scatter (FS) and 90° light scatter parameters (SS). For every histogram, a minimum of 20,000 events were counted. The mean fluorescence intensity was detected and expressed as a percentage of relative ROS level versus control cells.

### 2.10. Statistical Analysis

Data is presented as mean ± standard deviation (SD). Statistical analysis of the results was performed using Student's *t*-test, with GraphPad Prism v5.01 for Windows (GraphPad Software, San Diego, CA, USA). For all statistical tests, a two-tailed *p* value < 0.05 was considered significant. All data reported were verified in at least three independent experiments and expressed as mean ± SD.

## 3. Results

### 3.1. Synergy between AEs and Chemotherapeutic Drugs Induces Loss of Cancer Cell Viability

**Figure 1:**
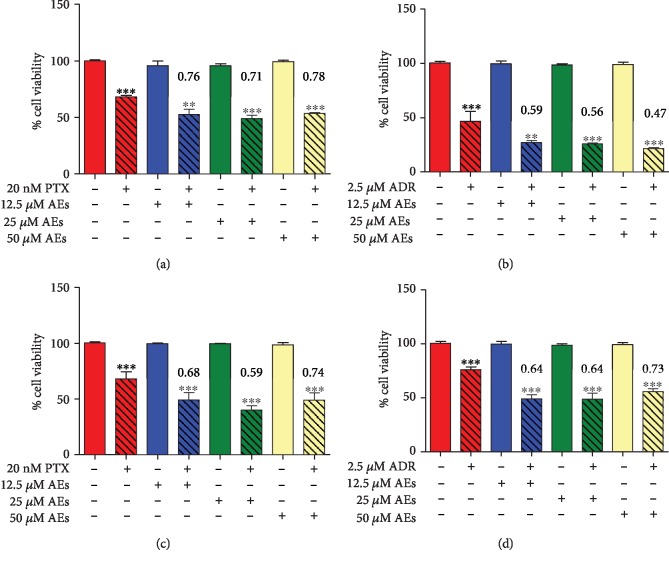
Effect of AEs on PTX or ADR-treated breast cancer cells. Cell viability assay: breast cancer cell lines (MDA-MB231 (a, b) and MCF7(c, d)) were treated with PTX (20 nM) or ADR (2.5 *μ*M) with or without AEs (from 12.5 to 50 *μ*M) for 24 h. Histograms show cell viability to highlight the effect of the association of chemotherapeutic agent and AEs. Synergy is characterized by a combination index < 0.8, and when present, its value is reported in red. Data is expressed as the mean ± SD of, at least, three independent experiments compared with a medium alone. The statistical significance between groups was calculated using Student's *t*-test. Significant differences are indicated by asterisks. MDA-MB231+PTX: PTX *vs.* 12.5 *μ*M AEs+PTX ^∗^*p* = 0.0127; PTX *vs.* 25 *μ*M AEs+PTX ^∗∗^*p* = 0.0037; PTX *vs.* 50 *μ*M AEs+PTX ^∗∗∗^*p* < 0.0001. MDA-MB231+ADR: ADR *vs.* 12.5 *μ*M AEs+ADR ^∗∗^*p* = 0.0070; ADR *vs.* 25 *μ*M AEs+ADR ^∗∗^*p* = 0.0049; ADR *vs.* 50 *μ*M AEs+ADR ^∗∗^*p* = 0.0019. MCF7+PTX: PTX *vs.* 12.5 *μ*M AEs+PTX ^∗∗^*p* = 0.0019; PTX *vs.* 25 *μ*M AEs+PTX ^∗∗∗^*p* < 0.0001; PTX *vs.* 50 *μ*M AEs+PTX ^∗∗^*p* = 0.0017. MCF7+ADR: ADR *vs.* 12.5 *μ*M AEs+ADR ^∗∗∗^*p* < 0.0001; ADR *vs.* 25 *μ*M AEs+ADR ^∗∗∗^*p* = 0.0001; ADR *vs.* 50 *μ*M AEs+ADR ^∗∗∗^*p* < 0.0001.

In order to highlight a potential effect of compound combination between AEs and the most active and widely used cancer drugs in clinical management (taxanes, anthracyclines, and platinum complexes), we used two breast cancer cell lines, MCF7 and MDA-MB231, as experimental models. In both cell lines, PTX, ADR, or CDDP was employed at fixed concentrations (20 nM, 2.0 *μ*M, and 20 *μ*M, respectively). These treatments for 24 h lead to a reduction of cell viability of 30% (IC 30). By using the algorithm described by Fransson et al. [51] to calculate the combination index (CI), we analyzed the effect of AEs plus PTX, ADR, and CDDP compared with that of the single agents. The addition of AEs, from 12.5 *μ*M up to 50 *μ*M, to PTX or ADR in MDA-MB231 (Figures 1(a) and 1(b)) and in MCF7 (Figured 1(c) and 1(d)) cells yielded a decrease in cell viability which could be ascribed to a chemosensitizing effect of AEs to these drugs. The analysis of this data showed that the combined treatment, as demonstrated by the CI values in Figure 1, enhances cytotoxicity in a synergistic manner. Conversely, the presence of AEs did not increase the number of dead cells caused by CDDP in a significant manner in both MCF7 and MDA-MB231 cells (Fig. [Supplementary-material supplementary-material-1]).

Since triple negative breast cancers have a more aggressive phenotype and a poorer prognosis due to the high propensity for metastatic progression and absence of specific hormonal-based targeted treatment, novel therapeutic strategies are required. To this end, we focused on MDA-MB231 as a TNBC cellular model to best describe a synergistic effect of AEs in combination with low doses of PTX, a first-line chemotherapeutic agent in breast cancer.

### 3.2. The Combination of AEs and PTX Inhibits Human Breast Cancer Cell Proliferation

In view of the aforementioned effects on cell viability, we thoroughly explored the mechanisms involved in the synergistic effect of the combined treatment on breast cancer cells.

To further characterize the decreased cell viability induced by cotreatment, we evaluated the expression of cleaved PARP (c-PARP) and LC3 as potential molecular signs of regulated cell death. No significant modulation of these proteins was detected compared to PTX treatment alone, which is well known to cause marked cell death [52, 53]. These slight effects that were detected demonstrate that both apoptotic and autophagic cell deaths are not relevant for the synergistic response of the combined treatment (Fig. [Supplementary-material supplementary-material-1]).

**Figure 2:**
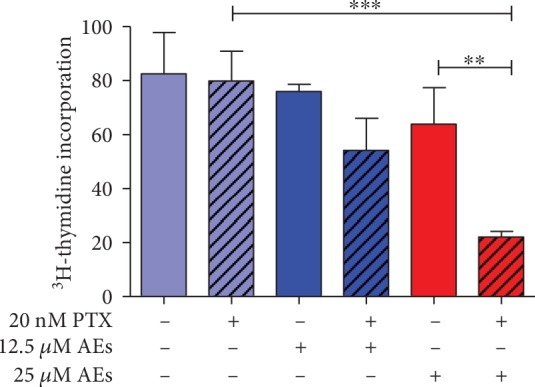
MDA-MB231 proliferation in response to AEs/PTX cotreatment. Proliferation assay: cells were exposed to AEs (12.5-25 *μ*M) plus and minus PTX (20 nM) for 24 h and the proliferation rate was measured by ^3^H-thymidine incorporation assay. PTX *vs.* 25 *μ*M AEs+PTX ^∗∗∗^*p* = 0.0009. 25 *μ*M AEs *vs.* 25 *μ*M AEs+PTX ^∗∗^*p* = 0.064.

These findings prompted us to detect DNA synthesis ratio in treated cells. As shown in Figure 2, AEs strongly decrease ^3^H-thymidine incorporation in a dose-dependent manner in MDA-MB231 cells treated with 20 nM PTX. The highest concentration tested (25 *μ*M AEs) inhibited cell proliferation by 70% compared to PTX alone.

In order to investigate additional features of cellular response to cotreatment, we evaluated the clonogenic ability of breast cancer cells. Since MDA-MB231 cells do not aggregate well and form very dispersed colonies [54], we switched to MCF7 cell model with a similar sensitivity to AEs/PTX (Figure 1) to test the effect of cotreatment on the colony formation ability. Our results demonstrate that this property was poorly affected (Fig. [Supplementary-material supplementary-material-1]).

### 3.3. Role of ROS in Synergistic Cytotoxicity in AE/PTX-Treated Cells

**Figure 3:**
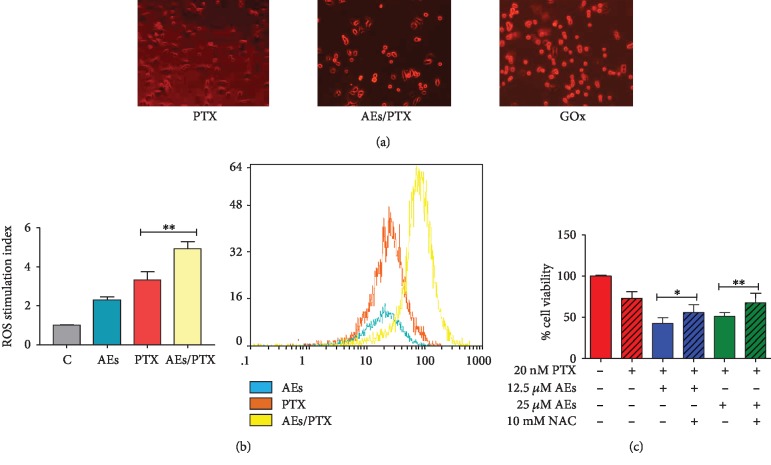
ROS production in MDA-MB231 cells treated with AEs/PTX. (a) Fluorescence microscopy: MDA-MB231 cells treated with PTX (20 nM) alone or AEs/PTX 25 *μ*M and 20 nM, respectively, or GOx (0.2 UI/ml) as a positive control for 24 h. The presence of ROS was detected by DHE fluorescent staining and red fluorescent-stained cells versus total cells were counted using an inverted fluorescence microscope (magnification 20x). (b, b1) Flow cytometry: the mean fluorescence intensity was expressed as stimulation index obtained by ratio between ROS levels released by cells after 2 h of treatment and ROS detection in control cells. Data is the mean ± SD of 3 independent experiments. Indicative fluorescence peaks of ROS production in cells after 2 h of treatment with 25 *μ*M AEs (blue graph), 20 nM PTX (orange graph), and AEs/PTX, respectively, 25 *μ*M and 20 nM (yellow graph) are reported in (b1). (c) Antioxidant effect on cell viability: NAC reduced the synergistic effect of AEs (12.5-25 *μ*M) in PTX (20 nM) treated cells. The cell viability results are the mean ± SD of at least three independent experiments. Significant statistical differences present in NAC plus and minus AE/PTX-treated cells are indicated by asterisks: 12.5 *μ*M AEs+PTX *vs*. 12.5 *μ*M AEs+PTX+NAC ^∗^*p* = 0.0191. 25 *μ*M AEs+PTX *vs*. 25 *μ*M AEs+PTX+NAC ^∗∗^*p* = 0.0094.

Based on the prooxidant activity of natural polyphenols in inducing cell growth inhibition [27, 55–57] and on the ability of PXT to promote intracellular ROS formation [32], we evaluated the oxidative pathway as a potential mechanism involved in AE/PTX-induced cell proliferation inhibition. In agreement with Chikara et al. data [58], which shows that several polyphenols strengthen the anticancer properties of chemotherapeutic drugs by elevating ROS levels, we report (Figure 3(a)) increased numbers of bright red fluorescent cells indicating enhanced levels of superoxide anions.

To further investigate the involvement of oxidative pathway in AE/PTX synergistic activity, the amount of ROS in particular peroxides was evaluated by flow cytometry. ROS production induced by the combined treatment takes place early, since it increases in relation to single agents after 2 h of exposure (Figures 3(b) and 3(b1)). This trend is less evident after 24 h treatment, probably as a result of ROS accumulation (Fig. [Supplementary-material supplementary-material-1]).

To further determine the role of ROS in AE/PTX-induced cell growth arrest, we sought to examine whether inhibition of ROS production by the well-known antioxidant NAC has any impact on synergy in breast cancer cell viability. As shown in Figure 3(c), the cell pretreatment of NAC significantly reduced the synergistic effect of 25 *μ*M AEs/PTX by about 20%.

### 3.4. Role of Nrf2/FEN1 and p-ERK/FEN1 Axis in DNA Damage Induced by AEs/PTX

**Figure 4:**
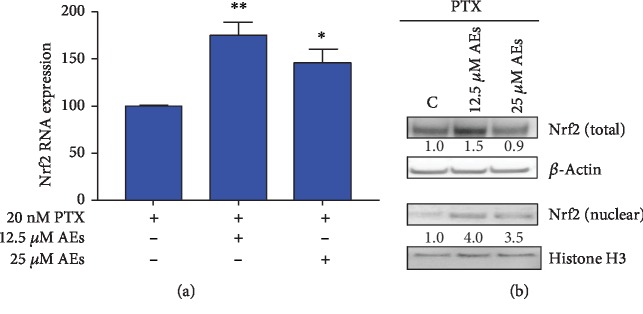
Nrf2 expression in MDA-MB231 treated with AEs/PTX. (a) Real-time assay. Nrf2 RNA expression is detected in cells treated with indicated concentrations of AEs plus and minus PTX (20 nM). 12.5 *μ*M AEs *vs.* 12.5 *μ*M AEs+PTX ^∗∗^*p* = 0.0023. 25 *μ*M AEs *vs.* 25 *μ*M AEs+PTX ^∗^*p* = 0.0176. (b) Nrf2 protein expression: total protein expression and nuclear translocation of Nrf2 were detected in treated cells. Quantification of band intensities was performed using ImageJ software, normalized by *β*-actin (total Nrf2) and histone H3 (nuclear Nrf2) expression levels. Relative values are calculated by comparing sample band intensities to control.

**Figure 5:**
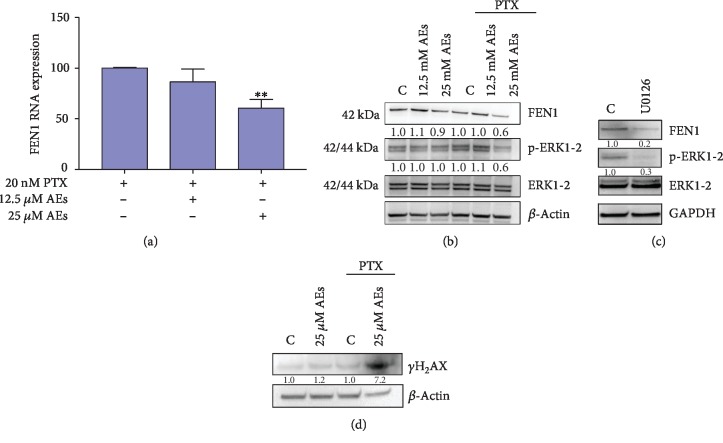
FEN1 modulation and DNA damage in combined treated MDA-MB231 cells. (a) Real-time assay: FEN1 RNA expression is detected in cells treated with indicated concentrations of AEs plus and minus PTX (20 nM). 25 *μ*M AEs *vs.* 25 *μ*M AEs+PTX ^∗∗^*p* = 0.0052. (b) FEN1/p-ERK1-2 protein expression: FEN1 protein expression and phosphorylation level of ERK1-2 were detected in total lysate of treated MDA-MB231 cells. Quantification of band intensities was performed using ImageJ software, normalized by *β*-actin expression level. Relative values are calculated by comparing sample band intensities to control in each setting (±PTX). (c) Effect of p-ERK1-2 inhibitor on FEN1 expression: FEN1 and p-ERK1-2 level were detected in MDA-MB231 treated for 60 minutes with U0126 (20 *μ*M), a MAPK/ERK inhibitor. Quantification of band intensities was performed using ImageJ software, normalized by GAPDH expression level. Relative values are calculated by comparing sample band intensities to control. (d) DNA damage level: DNA damage marker *γ*-H_2_AX was detected in cells treated with AEs plus and minus PTX. Quantification of band intensities was performed using ImageJ software, normalized by *β*-actin expression level. Relative values are calculated by comparing sample band intensities to control in each setting (±PTX).

After oxidative stress, Nrf2 is activated and moves to the nucleus where it regulates ARE transcriptional activity. Based on literature data [58–60], we hypothesize that combined treatment could affect Nrf2 activity. As shown in Figures 4(a) and 4(b), AEs increase the RNA/protein expression of Nrf2 in cells treated with PTX and induce a nuclear translocation of Nrf2 in this oxidative stress scenario. Since Nrf2 has been shown to be a repressor of the *Fen1* gene [61], we assessed the RNA expression of FEN1 in AE/PTX-treated cells. As reported in Figures 5(a) and 5(b), 25 *μ*M AEs induced a significant decrease in FEN1 RNA expression as well as in protein levels.

Since we detected a partial cytotoxicity rescue by NAC exposure, in order to explore the AE/PTX effect further, we hypothesized that a different molecular mechanism might be involved in the cellular synergistic response.

Several studies have reported that the MAPK family members play crucial roles in cell proliferation, survival, and differentiation [62, 63]. In particular, ERK kinases have been shown to play a part in DNA damage response (DDR) and that inhibition of p-ERK enhances the genotoxic effect of chemotherapeutic drugs [48]. Zou et al. reported that curcumin-treated breast cancer cells are more sensitive to cisplatin by downregulation of FEN1 achieved by reduction of p-ERK expression [19]. In agreement with published results, in our experimental settings, FEN1 and p-ERK expression levels decreased in combination treatment compared with PTX alone. Decrease in p-ERK was related to the chemosensitizing effect of 25 *μ*M AEs to PTX by targeting FEN1 in MDA-MB231 cells (Figure 5(b)). In order to show a direct correlation between p-ERK and FEN1 expression, untreated MDA-MB231 cells were exposed to ERK inhibitor U0126 (20 *μ*M). After 60 minutes of U0126 exposure, both ERK phosphorylation and FEN1 expression were clearly downregulated (Figure 5(c)).

To test DNA damage level related to FEN1 downregulation, we looked at the extent of H_2_AX phosphorylation (*γ*-H_2_AX), a sensitive indicator of DNA double strand breaks (Figure 5(d)). We detected a marked phosphorylation level of histone H_2_AX in the experimental cellular setting treated with 25 *μ*M AEs plus PTX, compared to PTX alone.

This data is evidence that AEs strengthen the antitumor activity of PTX both through the ROS/Nrf2 pathway and *via* the downregulation of p-ERK, which result in the decrease in FEN1 expression.

These findings indicate that the synergistic response is a result of balance between the ROS/Nrf2 pathway and the impaired DNA damage response (DDR) triggered by FEN1 downregulation, suggesting the presence of at least two molecular mechanisms involved in the synergistic cytotoxicity elicited by our combined treatment.

## 4. Discussion

To explore the role of natural compounds as chemosensitizer agents in breast cancer, we have investigated the effect of combined treatment of artichoke polyphenolic extracts with paclitaxel, adriamycin, or cisplatin on breast cancer cell lines. We demonstrate that AEs synergized with PTX or ADR in hindering the growth of MDA-MB231 or MCF7 cells compared with drug alone. AEs enhanced breast cancer sensitivity to PTX by decreasing cell proliferation both through the ROS/Nrf2 pathway and *via* the downregulation of p-ERK, and these mechanisms resulted in the decrease in FEN1 expression leading to DNA damage accumulation and DNA replication reduction. These findings suggest the presence of at least two molecular mechanisms involved in the synergistic AE/PTX cytotoxicity in breast cancer cells.

Tumor cells exhibit excessive ROS production which is related to aberrant metabolism and continuous cell division; therefore, cancer cells appear to be more vulnerable to further oxidative insult compared to normal ones [64]. ROS cellular levels are thus crucial for designing advanced therapies and a future challenge in anticancer treatment [58]. There are many reports describing the prooxidant effect of polyphenols in sensitizing cancer cells to chemotherapeutic drugs through different pathways [38–41, 65–67].

In light of our previous findings regarding the role of AEs as prooxidant players on breast cancer cells [27], this study has explored the ability of AEs to sensitize breast cancer cell to conventional chemotherapy through oxidative process. Based on literature data [38–42], our results strongly support the prooxidant role of AEs/PTX in hindering breast cancer cellular growth associated with ROS production. To further confirm the important role of ROS in enhancing the antitumor effect of the combined treatment, we demonstrate that the well-known antioxidant NAC attenuates the synergistic and antitumor effect on MDA-MB231 cells. Since we detected a partial cytotoxicity rescue by NAC exposure, we hypothesized that a further molecular mechanism was involved in the cellular synergistic response.

In agreement with published results, our data has shown that many anticancer drugs including ADR and PTX but not CDDP have the ability to induce oxidative stress [32]. We have shown that both ADR and PTX plus and minus AEs induce a synergistic effect on reducing cell viability, while AE/CDDP combined treatment did not modify cell death ratio. This finding suggests that AEs are able to produce ROS and synergize with the prooxidant activity of some chemotherapeutic drugs, which suggests that at least one of the mechanism involved in synergistic interaction is ROS production dependent.

Nrf2 is a main transcription factor in the regulation of many phase II and antioxidant genes [43, 44]. It is well known that induction of phase II enzymes, counteracting reactive electrophiles including ROS, plays an important role in response to many chemotherapeutic agents. Nrf2 is located in the cytoplasm in a complex with the actin-binding protein Keap1. Upon oxidative stimulation, Nrf2 moves to the nucleus where it interacts with AREs present in the promoter region of many genes [45]. In our experimental model, after AE/PTX exposure for 24 h, the Nrf2 mRNA level, together with the nuclear protein level, significantly increased compared with PTX alone treatment. According to Kwak et al. [68], who demonstrated that Nrf2 has a short half-life, we hypothesized that the nuclear accumulation is due to *de novo* synthesis of the transcription factor. In agreement with Chen et al. [61], who showed that Nrf2 bounds to ARE-like sequence located in the FEN1 promoter region, we observed that Nrf2 downregulated the expression of FEN1, which suggests that Nrf2 may function as a repressor of this endonuclease gene.

Our data has shown that cotreatment significantly reduced the FEN1 RNA level together with a downregulation of the protein expression compared with PTX alone and suggests that such a mechanism is involved in the antiproliferative effect of AEs/PTX. The combined treatment results in DNA damage accumulation, confirmed by the phosphorylation of H_2_AX which leads to DNA replication reduction. This data suggests that downregulating the expression of FEN1, a potential biomarker and therapeutic target [36, 69], may be a new mechanism by which AEs sensitize breast cancer cells to PTX.

It has been shown that polyphenols can modulate multiple signaling pathways including MAPK/ERK [19, 38, 70, 71]. By inhibiting ERK phosphorylation, curcumin helps to sensitize the cells to cisplatin by targeting FEN1 [19]. In agreement with this data, our results showed that FEN1 and p-ERK expression levels decreased in combination treatment compared with PTX alone. Since the inhibition of ERK activation by the common MEK inhibitor U0126 induced a downregulation of FEN1, we envisage a marked correlation between this nuclease and the aforementioned kinase expression level in our experiments. As previously reported, aberrant activation of the ERK pathway is one of the most relevant events in human cancer as it stimulates cell proliferation [72]. However, the activation of the ERK pathway has an important role in DDR and has been associated with chemotherapeutic drugs commonly considered to be DNA damage inducers. For several tumors, inhibition of MAPK/ERK cascade could enhance the genotoxic effect of chemotherapeutic agents. This is probably because of the accumulation of DNA lesions due to the impaired checkpoint activation ERK related [48]. Therefore, if a combinatory therapy is to be used, it is important to determine if the combination will lead to inefficient repair of DNA lesions due to downregulation of FEN1.

Our results summarized in supplementary materials (Fig. [Supplementary-material supplementary-material-1]) suggest that artichoke polyphenols can be used as sensitizers of chemotherapy paving the way for new combined treatment in oncology. Since polyphenols are widely used as dietary components and have shown no toxicity in humans, their use as adjuvant agents may be an innovative and promising natural compound-based therapeutic strategy. Our study adds a novel aspect of the underlying mechanisms of the anticancer properties of AEs to our previous findings. However, wide-ranging pharmacokinetic and metabolic studies of combined treatment on animal models are required to evaluate its efficacy in human clinical trials.

## 5. Conclusions

We envisage a combined AE/chemotherapeutic agent treatment that is able to reduce the dose of antitumor drugs minimizing toxicity and side effects of conventional cancer therapy.

## Data Availability

The data used to support the findings of this study is included within the article.

## Supplementary Materials

Supplementary Materials Supplementary materials integrate data described in the main document. Fig. 1s: effect of AEs on CDDP treated breast cancer cells. Cell viability assay: MDA-MB231 (a) and MCF7 (b) were treated with CDDP (20 *μ*M) with or without AEs (from 12.5 to 50 *μ*M) for 24 h. Histograms show cell viability. No significant differences are detected between combined treatment vs. CDDP alone. Fig. 2s: effect of AEs/PTX on regulated cell death. Apoptosis and autophagy marker analysis: cells were incubated with AEs or AEs/PTX and then assayed via western blot for c-PARP (a) and LC3 (b). Fig. 3s: cloning efficiency. Colony-forming analysis: MCF7 cells were exposed to AEs or AEs/PTX for 24 h and then allowed to grow and form colonies for subsequent 14 d. Cell colonies, after staining with crystal violet (a), were counted and the values reported as percent colony number in the histogram (b). C vs. PTX ^∗∗^*p* = 0.0049, 12.5 *μ*M AEs vs. 12.5 *μ*M AEs/PTX ^∗∗∗^*p* < 0.0001, and 25 *μ*M AEs vs. 25 *μ*M AEs/PTX ^∗∗∗^*p* = 0.0005. There is no significant difference between colony numbers of cells treated with AEs/PTX or PTX alone. Fig. 4s: ROS production in MDA-MB231 cells after 24 h of treatment. (a, a1) Flow cytometry: the mean fluorescence intensity was expressed as stimulation index obtained by ratio between ROS levels released by cells after 24 h of treatment and ROS detection in control cells. Data is the mean ± SD of 3 independent experiments. Indicative fluorescence peaks of ROS production in cells after 24 h of treatment with 25 *μ*M AEs (red graph), 20 nM PTX (purple graph), and AEs/PTX, respectively, 25 *μ*M and 20 nM (brown graph) are reported in (a1). There is no significant difference between groups. Fig. 5s: graphical abstract. Schematic representation of two convergent signaling pathways involved in synergistic effect of AE/PTX combined treatment. The figure suggests the potential role of artichoke polyphenols as sensitizers of chemotherapy in breast cancer cells.

## References

[1] Ghoncheh M., Mohammadian M., Mohammadian-Hafshejani A., Salehiniya H. (2016). The incidence and mortality of colorectal cancer and its relationship with the human development index in Asia. *Annals of Global Health*.

[2] Martelotto L. G., Ng C. K. Y., Piscuoglio S., Weigelt B., Reis-Filho J. S. (2014). Breast cancer intra-tumor heterogeneity. *Breast Cancer Research*.

[3] Song J. L., Chen C., Yuan J. P., Sun S. R. (2016). Progress in the clinical detection of heterogeneity in breast cancer. *Cancer Medicine*.

[4] Zardavas D., Irrthum A., Swanton C., Piccart M. (2015). Clinical management of breast cancer heterogeneity. *Nature Reviews. Clinical Oncology*.

[5] Prat A., Fan C., Fernández A. (2015). Response and survival of breast cancer intrinsic subtypes following multi-agent neoadjuvant chemotherapy. *BMC Medicine*.

[6] Goto W., Kashiwagi S., Takada K. (2018). Significance of intrinsic breast cancer subtypes on the long-term prognosis after neoadjuvant chemotherapy. *Journal of Translational Medicine*.

[7] Bayat Mokhtari R., Homayouni T. S., Baluch N. (2017). Combination therapy in combating cancer. *Oncotarget*.

[8] Farvid M. S., Chen W. Y., Rosner B. A., Tamimi R. M., Willett W. C., Eliassen A. H. (2019). Fruit and vegetable consumption and breast cancer incidence: repeated measures over 30 years of follow-up. *International Journal of Cancer*.

[9] Rothwell J. A., Knaze V., Zamora-Ros R. (2017). Polyphenols: dietary assessment and role in the prevention of cancers. *Current Opinion in Clinical Nutrition and Metabolic Care*.

[10] Braakhuis A., Campion P., Bishop K. (2016). Reducing breast cancer recurrence: the role of dietary polyphenolics. *Nutrients*.

[11] Dandawate P. R., Subramaniam D., Jensen R. A., Anant S. (2016). Targeting cancer stem cells and signaling pathways by phytochemicals: novel approach for breast cancer therapy. *Seminars in Cancer Biology*.

[12] Eitsuka T., Tatewaki N., Nishida H., Nakagawa K., Miyazawa T. (2016). Synergistic anticancer effect of tocotrienol combined with chemotherapeutic agents or dietary components: a review. *International Journal of Molecular Sciences*.

[13] Burnett J. P., Lim G., Li Y. (2017). Sulforaphane enhances the anticancer activity of taxanes against triple negative breast cancer by killing cancer stem cells. *Cancer Letters*.

[14] Al-Abbasi F., Alghamdi E., Baghdadi M. (2016). Gingerol synergizes the cytotoxic effects of doxorubicin against liver cancer cells and protects from its vascular toxicity. *Molecules*.

[15] Kapadia G. J., Rao G. S., Ramachandran C., Iida A., Suzuki N., Tokuda H. (2013). Synergistic cytotoxicity of red beetroot (Beta vulgaris L.) extract with doxorubicin in human pancreatic, breast and prostate cancer cell lines. *Journal of Complementary and Integrative Medicine*.

[16] Chatterjee S., Rhee Y. H., Ahn J. C. (2016). Sulforaphene-carboplatin combination synergistically enhances apoptosis by disruption of mitochondrial membrane potential and cell cycle arrest in human non-small cell lung carcinoma. *Journal of Medicinal Food*.

[17] Hossain M. M., Banik N. L., Ray S. K. (2012). Synergistic anti-cancer mechanisms of curcumin and paclitaxel for growth inhibition of human brain tumor stem cells and LN18 and U138MG cells. *Neurochemistry International*.

[18] Piccolo M. T., Menale C., Crispi S. (2015). Combined anticancer therapies: an overview of the latest applications. *Anti-Cancer Agents in Medicinal Chemistry*.

[19] Zou J., Zhu L., Jiang X. (2018). Curcumin increases breast cancer cell sensitivity to cisplatin by decreasing FEN1 expression. *Oncotarget*.

[20] Muthusamy G., Balupillai A., Ramasamy K. (2016). Ferulic acid reverses ABCB1-mediated paclitaxel resistance in MDR cell lines. *European Journal of Pharmacology*.

[21] Saénz Rodriguez T., García Giménez D., de la Puerta Vázquez R. (2002). Choleretic activity and biliary elimination of lipids and bile acids induced by an artichoke leaf extract in rats. *Phytomedicine*.

[22] Gebhardt R. (1998). Inhibition of cholesterol biosynthesis in primary cultured rat hepatocytes by artichoke (Cynara scolymus L.) extracts. *The Journal of Pharmacology and Experimental Therapeutics*.

[23] Wider B., Pittler M. H., Thompson-Coon J., Ernst E. (2013). Artichoke leaf extract for treating hypercholesterolaemia. *Cochrane Database of Systematic Reviews*.

[24] Miccadei S., Di Venere D., Cardinali A. (2008). Antioxidative and apoptotic properties of polyphenolic extracts from edible part of artichoke (Cynara scolymus L.) on cultured rat hepatocytes and on human hepatoma cells. *Nutrition and Cancer*.

[25] Mileo A. M., Di Venere D., Linsalata V., Fraioli R., Miccadei S. (2012). Artichoke polyphenols induce apoptosis and decrease the invasive potential of the human breast cancer cell line MDA-MB231. *Journal of Cellular Physiology*.

[26] Pulito C., Mori F., Sacconi A. (2015). Cynara scolymus affects malignant pleural mesothelioma by promoting apoptosis and restraining invasion. *Oncotarget*.

[27] Mileo A. M., Di Venere D., Abbruzzese C., Miccadei S. (2015). Long Term Exposure to Polyphenols of Artichoke (*Cynara scolymus* L.) Exerts Induction of Senescence Driven Growth Arrest in the MDA-MB231 Human Breast Cancer Cell Line. *Oxidative Medicine and Cellular Longevity*.

[28] Azzini E., Bugianesi R., Romano F. (2007). Absorption and metabolism of bioactive molecules after oral consumption of cooked edible heads of Cynara scolymus L. (cultivar Violetto di Provenza) in human subjects: a pilot study. *The British Journal of Nutrition*.

[29] King K. M., Lupichuk S., Baig L. (2009). Optimal use of taxanes in metastatic breast cancer. *Current Oncology*.

[30] Xiao H., Verdier-Pinard P., Fernandez-Fuentes N. (2006). Insights into the mechanism of microtubule stabilization by Taxol. *Proceedings of the National Academy of Sciences of the United States of America*.

[31] McGrogan B. T., Gilmartin B., Carney D. N., McCann A. (2008). Taxanes, microtubules and chemoresistant breast cancer. *Biochimica et Biophysica Acta*.

[32] Yokoyama C., Sueyoshi Y., Ema M., Mori Y., Takaishi K., Hisatomi H. (2017). Induction of oxidative stress by anticancer drugs in the presence and absence of cells. *Oncology Letters*.

[33] Balakrishnan L., Bambara R. A. (2013). Flap endonuclease 1. *Annual Review of Biochemistry*.

[34] Liu Y., Kao H. I., Bambara R. A. (2004). Flap endonuclease 1: a central component of DNA metabolism. *Annual Review of Biochemistry*.

[35] Bambara R. A., Murante R. S., Henricksen L. A. (1997). Enzymes and reactions at the eukaryotic DNA replication fork. *The Journal of Biological Chemistry*.

[36] Zheng L., Jia J., Finger L. D., Guo Z., Zer C., Shen B. (2011). Functional regulation of FEN1 nuclease and its link to cancer. *Nucleic Acids Research*.

[37] Birben E., Sahiner U. M., Sackesen C., Erzurum S., Kalayci O. (2012). Oxidative stress and antioxidant defense. *World Allergy Organization Journal*.

[38] Park B. H., Lim J. E., Jeon H. G. (2016). Curcumin potentiates antitumor activity of cisplatin in bladder cancer cell lines via ROS-mediated activation of ERK1/2. *Oncotarget*.

[39] Huang Y. F., Zhu D. J., Chen X. W. (2017). Curcumin enhances the effects of irinotecan on colorectal cancer cells through the generation of reactive oxygen species and activation of the endoplasmic reticulum stress pathway. *Oncotarget*.

[40] Nie P., Hu W., Zhang T., Yang Y., Hou B., Zou Z. (2015). Synergistic induction of erlotinib-mediated apoptosis by resveratrol in human non-small-cell lung cancer cells by down-regulating survivin and up-regulating PUMA. *Cellular Physiology and Biochemistry*.

[41] Mondal A., Bennett L. L. (2016). Resveratrol enhances the efficacy of sorafenib mediated apoptosis in human breast cancer MCF7 cells through ROS, cell cycle inhibition, caspase 3 and PARP cleavage. *Biomedicine & Pharmacotherapy*.

[42] Chan M. M., Soprano K. J., Weinstein K., Fong D. (2006). Epigallocatechin-3-gallate delivers hydrogen peroxide to induce death of ovarian cancer cells and enhances their cisplatin susceptibility. *Journal of Cellular Physiology*.

[43] Kwak M. K., Itoh K., Yamamoto M., Sutter T. R., Kensler T. W. (2001). Role of transcription factor Nrf2 in the induction of hepatic phase 2 and antioxidative enzymes in vivo by the cancer chemoprotective agent, 3H-1, 2-dimethiole-3-thione. *Molecular Medicine*.

[44] Ramos-Gomez M., Kwak M. K., Dolan P. M. (2001). Sensitivity to carcinogenesis is increased and chemoprotective efficacy of enzyme inducers is lost in nrf2 transcription factor-deficient mice. *Proceedings of the National Academy of Sciences of the United States of America*.

[45] Taguchi K., Yamamoto M. (2017). The KEAP1-NRF2 system in cancer. *Frontiers in Oncology*.

[46] Steelman L. S., Pohnert S. C., Shelton J. G., Franklin R. A., Bertrand F. E., McCubrey J. (2004). JAK/STAT, Raf/MEK/ERK, PI3K/Akt and BCR-ABL in cell cycle progression and leukemogenesis. *Leukemia*.

[47] McCubrey J. A., Steelman L. S., Chappell W. H. (2007). Roles of the Raf/MEK/ERK pathway in cell growth, malignant transformation and drug resistance. *Biochimica et Biophysica Acta (BBA) - Molecular Cell Research*.

[48] Wei F., Yan J., Tang D. (2011). Extracellular signal-regulated kinases modulate DNA damage response - a contributing factor to using MEK inhibitors in cancer therapy. *Current Medicinal Chemistry*.

[49] Livak K. J., Schmittgen T. D. (2001). Analysis of relative gene expression data using real-time quantitative PCR and the 2(-delta delta C(T)) method. *Methods*.

[50] Mari E., Mardente S., Morgante E. (2016). Graphene oxide nanoribbons induce autophagic vacuoles in neuroblastoma cell lines. *International Journal of Molecular Sciences*.

[51] Fransson A., Glaessgen D., Alfredsson J., Wiman K. G., Bajalica-Lagercrantz S., Mohell N. (2016). Strong synergy with APR-246 and DNA-damaging drugs in primary cancer cells from patients with TP53 mutant high-grade serous ovarian cancer. *Journal of Ovarian Research*.

[52] Calaf G. M., Ponce-Cusi R., Carrion F. (2018). Curcumin and paclitaxel induce cell death in breast cancer cell lines. *Oncology Reports*.

[53] McCloskey D. E., Kaufmann S. H., Prestigiacomo L. J., Davidson N. E. (1996). Paclitaxel induces programmed cell death in MDA-MB-468 human breast cancer cells. *Clinical Cancer Research*.

[54] Tseng C. N., Hong Y. R., Chang H. W. (2014). Brefeldin A reduces anchorage-independent survival, cancer stem cell potential and migration of MDA-MB-231 human breast cancer cells. *Molecules*.

[55] Halliwell B. (2008). Are polyphenols antioxidants or pro-oxidants? What do we learn from cell culture and in vivo studies?. *Archives of Biochemistry and Biophysics*.

[56] Kim H. S., Quon M. J., Kim J. A. (2014). New insights into the mechanisms of polyphenols beyond antioxidant properties; lessons from the green tea polyphenol, epigallocatechin 3-gallate. *Redox Biology*.

[57] Eghbaliferiz S., Iranshahi M. (2016). Prooxidant activity of polyphenols, flavonoids, anthocyanins and carotenoids: updated review of mechanisms and catalyzing metals. *Phytotherapy Research*.

[58] Chikara S., Nagaprashantha L. D., Singhal J., Horne D., Awasthi S., Singhal S. S. (2018). Oxidative stress and dietary phytochemicals: role in cancer chemoprevention and treatment. *Cancer Letters*.

[59] Enkhbat T., Nishi M., Yoshikawa K. (2018). Epigallocatechin-3-gallate enhances radiation sensitivity in colorectal cancer cells through Nrf2 activation and autophagy. *Anticancer Research*.

[60] Velavan B., Divya T., Sureshkumar A., Sudhandiran G. (2018). Nano-chemotherapeutic efficacy of (-) -epigallocatechin 3-gallate mediating apoptosis in A549 cells: involvement of reactive oxygen species mediated Nrf2/Keap1signaling. *Biochemical and Biophysical Research Communications*.

[61] Chen B., Zhang Y., Wang Y., Rao J., Jiang X., Xu Z. (2014). Curcumin inhibits proliferation of breast cancer cells through Nrf2-mediated down-regulation of Fen1 expression. *The Journal of Steroid Biochemistry and Molecular Biology*.

[62] Deschênes-Simard X., Kottakis F., Meloche S., Ferbeyre G. (2014). ERKs in cancer: friends or foes?. *Cancer Research*.

[63] Shaul Y. D., Seger R. (2007). The MEK/ERK cascade: from signaling specificity to diverse functions. *Biochimica et Biophysica Acta*.

[64] Trachootham D., Lu W., Ogasawara M. A., Valle N. R. D., Huang P. (2008). Redox regulation of cell survival. *Antioxidants & Redox Signaling*.

[65] Luo H., Yang A., Schulte B. A., Wargovich M. J., Wang G. Y. (2013). Resveratrol induces premature senescence in lung cancer cells via ROS-mediated DNA damage. *PLoS One*.

[66] Wu W. J., Zhang Y., Zeng Z. L. (2013). *β*-Phenylethyl isothiocyanate reverses platinum resistance by a GSH-dependent mechanism in cancer cells with epithelial-mesenchymal transition phenotype. *Biochemical Pharmacology*.

[67] Li Q., Zhan M., Chen W. (2016). Phenylethyl isothiocyanate reverses cisplatin resistance in biliary tract cancer cells via glutathionylation-dependent degradation of Mcl-1. *Oncotarget*.

[68] Kwak M. K., Itoh K., Yamamoto M., Kensler T. W. (2002). Enhanced expression of the transcription factor Nrf2 by cancer chemopreventive agents: role of antioxidant response element-like sequences in the nrf2 promoter. *Molecular and Cellular Biology*.

[69] Singh P., Yang M., Dai H. (2008). Overexpression and hypomethylation of flap endonuclease 1 gene in breast and other cancers. *Molecular Cancer Research*.

[70] Yan X., Qi M., Li P., Zhan Y., Shao H. (2017). Apigenin in cancer therapy: anti-cancer effects and mechanisms of action. *Cell & Bioscience*.

[71] Lei M. J., Dong Y., Sun C. X., Zhang X. H. (2017). Resveratrol inhibits proliferation, promotes differentiation and melanogenesis in HT-144 melanoma cells through inhibition of MEK/ERK kinase pathway. *Microbial Pathogenesis*.

[72] Montagut C., Settleman J. (2009). Targeting the RAF-MEK-ERK pathway in cancer therapy. *Cancer Letters*.

