# Supplementary figures and images for: Artichoke Polyphenols Sensitize Human Breast Cancer Cells to Chemotherapeutic Drugs via a ROS-Mediated Downregulation of Flap Endonuclease 1
Source: Oxid Med Cell Longev. 2020 Jan 3;2020:7965435. doi: 10.1155/2020/7965435 (PMC6969650; doi:10.1155/2020/7965435)

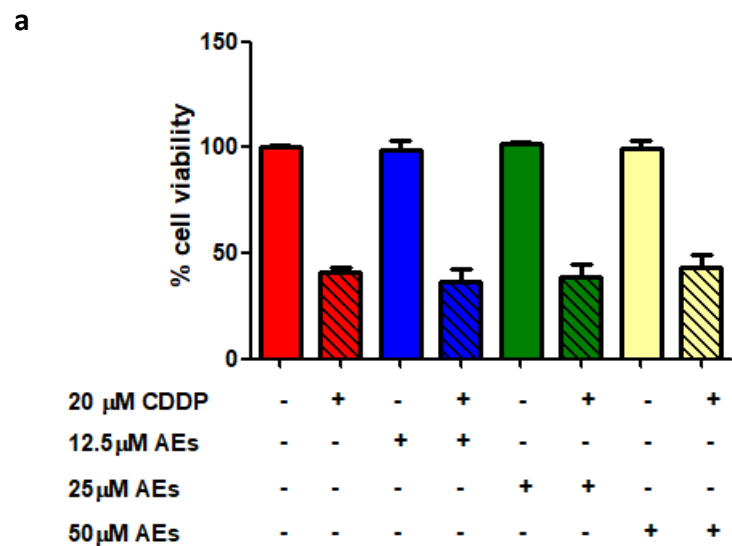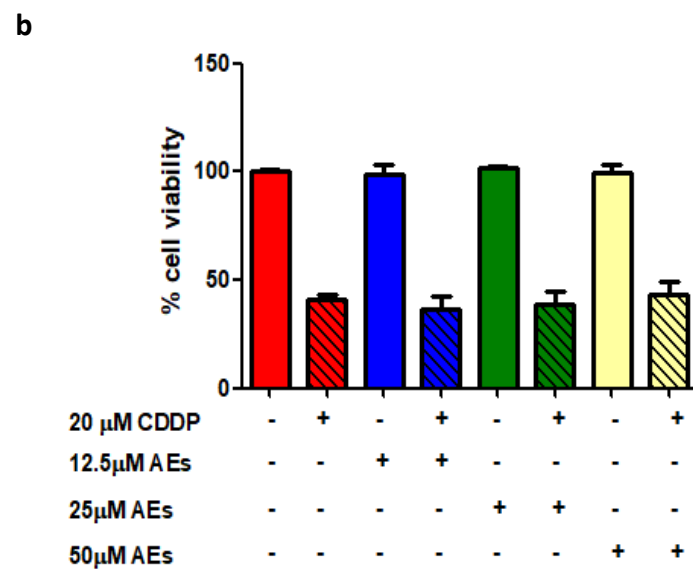

**Fig.1s**

Supplement: Supplementary Materials — integrate data described in the main document. Fig. 1s: effect of AEs on CDDP treated breast cancer cells. Cell viability assay: MDA-MB231 (a) and MCF7 (b) were treated with CDDP (20 μM) with or without AEs (from 12.5 to 50 μM) for 24 h. Histograms show cell viability. No significant differences are detected between combined treatment vs. CDDP alone. Fig. 2s: effect of AEs/PTX on regulated cell death. Apoptosis and autophagy marker analysis: cells were incubated with AEs or AEs/PTX and then assayed via western blot for c-PARP (a) and LC3 (b). Fig. 3s: cloning efficiency. Colony-forming analysis: MCF7 cells were exposed to AEs or AEs/PTX for 24 h and then allowed to grow and form colonies for subsequent 14 d. Cell colonies, after staining with crystal violet (a), were counted and the values reported as percent colony number in the histogram (b). C vs. PTX ∗∗p = 0.0049, 12.5 μM AEs vs. 12.5 μM AEs/PTX ∗∗∗p < 0.0001, and 25 μM AEs vs. 25 μM AEs/PTX ∗∗∗p = 0.0005. There is no significant difference between colony numbers of cells treated with AEs/PTX or PTX alone. Fig. 4s: ROS production in MDA-MB231 cells after 24 h of treatment. (a, a1) Flow cytometry: the mean fluorescence intensity was expressed as stimulation index obtained by ratio between ROS levels released by cells after 24 h of treatment and ROS detection in control cells. Data is the mean ± SD of 3 independent experiments. Indicative fluorescence peaks of ROS production in cells after 24 h of treatment with 25 μM AEs (red graph), 20 nM PTX (purple graph), and AEs/PTX, respectively, 25 μM and 20 nM (brown graph) are reported in (a1). There is no significant difference between groups. Fig. 5s: graphical abstract. Schematic representation of two convergent signaling pathways involved in synergistic effect of AE/PTX combined treatment. The figure suggests the potential role of artichoke polyphenols as sensitizers of chemotherapy in breast cancer cells. [file 7965435.f1.zip › Fig 1s .pdf]

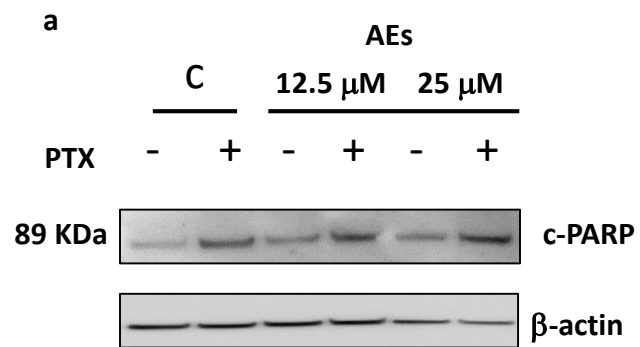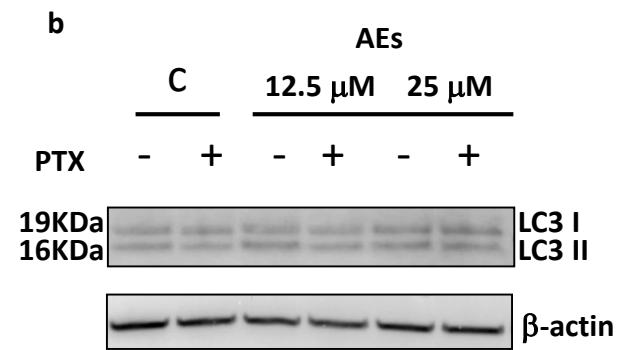

**Fig. 2s**

Supplement: Supplementary Materials — integrate data described in the main document. Fig. 1s: effect of AEs on CDDP treated breast cancer cells. Cell viability assay: MDA-MB231 (a) and MCF7 (b) were treated with CDDP (20 μM) with or without AEs (from 12.5 to 50 μM) for 24 h. Histograms show cell viability. No significant differences are detected between combined treatment vs. CDDP alone. Fig. 2s: effect of AEs/PTX on regulated cell death. Apoptosis and autophagy marker analysis: cells were incubated with AEs or AEs/PTX and then assayed via western blot for c-PARP (a) and LC3 (b). Fig. 3s: cloning efficiency. Colony-forming analysis: MCF7 cells were exposed to AEs or AEs/PTX for 24 h and then allowed to grow and form colonies for subsequent 14 d. Cell colonies, after staining with crystal violet (a), were counted and the values reported as percent colony number in the histogram (b). C vs. PTX ∗∗p = 0.0049, 12.5 μM AEs vs. 12.5 μM AEs/PTX ∗∗∗p < 0.0001, and 25 μM AEs vs. 25 μM AEs/PTX ∗∗∗p = 0.0005. There is no significant difference between colony numbers of cells treated with AEs/PTX or PTX alone. Fig. 4s: ROS production in MDA-MB231 cells after 24 h of treatment. (a, a1) Flow cytometry: the mean fluorescence intensity was expressed as stimulation index obtained by ratio between ROS levels released by cells after 24 h of treatment and ROS detection in control cells. Data is the mean ± SD of 3 independent experiments. Indicative fluorescence peaks of ROS production in cells after 24 h of treatment with 25 μM AEs (red graph), 20 nM PTX (purple graph), and AEs/PTX, respectively, 25 μM and 20 nM (brown graph) are reported in (a1). There is no significant difference between groups. Fig. 5s: graphical abstract. Schematic representation of two convergent signaling pathways involved in synergistic effect of AE/PTX combined treatment. The figure suggests the potential role of artichoke polyphenols as sensitizers of chemotherapy in breast cancer cells. [file 7965435.f1.zip › Fig 2s.pdf]

A

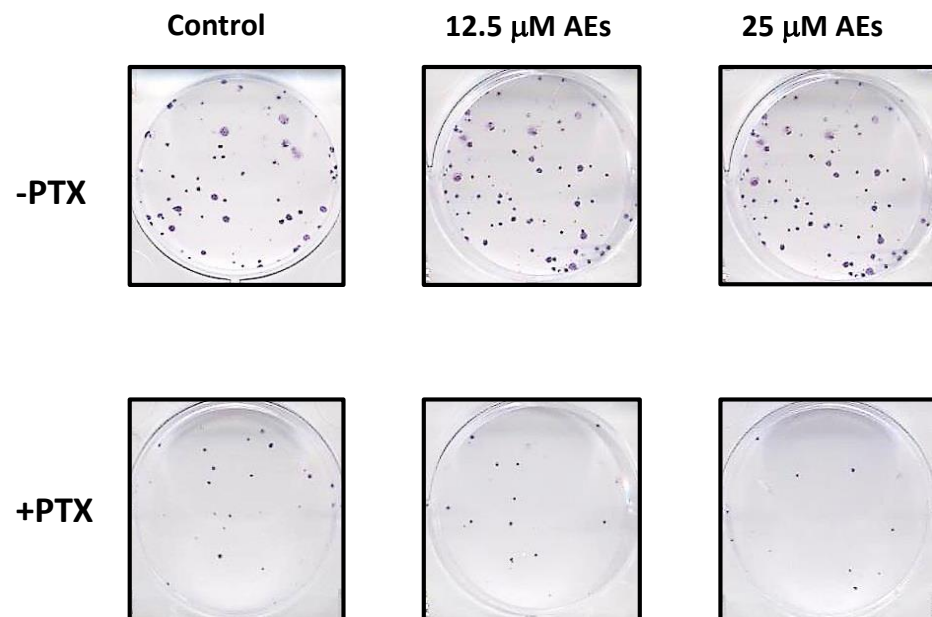

B

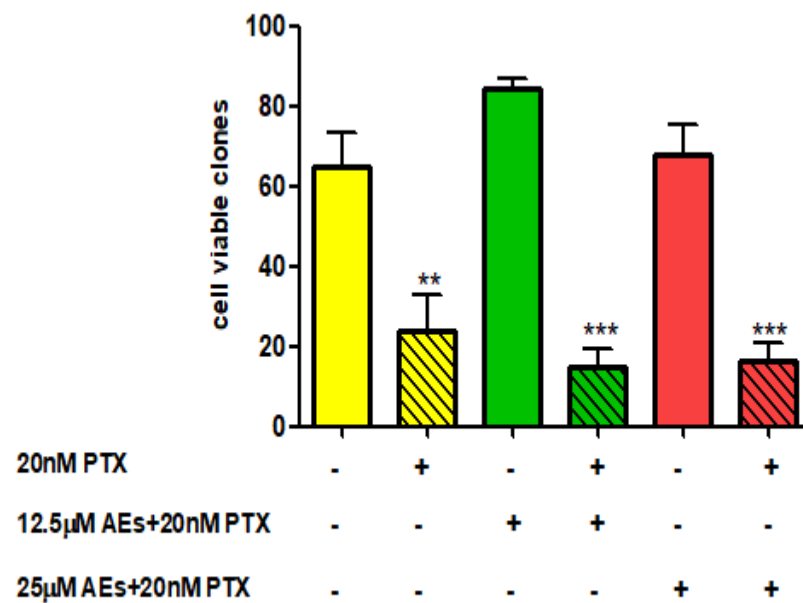

Fig.3s

Supplement: Supplementary Materials — integrate data described in the main document. Fig. 1s: effect of AEs on CDDP treated breast cancer cells. Cell viability assay: MDA-MB231 (a) and MCF7 (b) were treated with CDDP (20 μM) with or without AEs (from 12.5 to 50 μM) for 24 h. Histograms show cell viability. No significant differences are detected between combined treatment vs. CDDP alone. Fig. 2s: effect of AEs/PTX on regulated cell death. Apoptosis and autophagy marker analysis: cells were incubated with AEs or AEs/PTX and then assayed via western blot for c-PARP (a) and LC3 (b). Fig. 3s: cloning efficiency. Colony-forming analysis: MCF7 cells were exposed to AEs or AEs/PTX for 24 h and then allowed to grow and form colonies for subsequent 14 d. Cell colonies, after staining with crystal violet (a), were counted and the values reported as percent colony number in the histogram (b). C vs. PTX ∗∗p = 0.0049, 12.5 μM AEs vs. 12.5 μM AEs/PTX ∗∗∗p < 0.0001, and 25 μM AEs vs. 25 μM AEs/PTX ∗∗∗p = 0.0005. There is no significant difference between colony numbers of cells treated with AEs/PTX or PTX alone. Fig. 4s: ROS production in MDA-MB231 cells after 24 h of treatment. (a, a1) Flow cytometry: the mean fluorescence intensity was expressed as stimulation index obtained by ratio between ROS levels released by cells after 24 h of treatment and ROS detection in control cells. Data is the mean ± SD of 3 independent experiments. Indicative fluorescence peaks of ROS production in cells after 24 h of treatment with 25 μM AEs (red graph), 20 nM PTX (purple graph), and AEs/PTX, respectively, 25 μM and 20 nM (brown graph) are reported in (a1). There is no significant difference between groups. Fig. 5s: graphical abstract. Schematic representation of two convergent signaling pathways involved in synergistic effect of AE/PTX combined treatment. The figure suggests the potential role of artichoke polyphenols as sensitizers of chemotherapy in breast cancer cells. [file 7965435.f1.zip › Fig 3s .pdf]

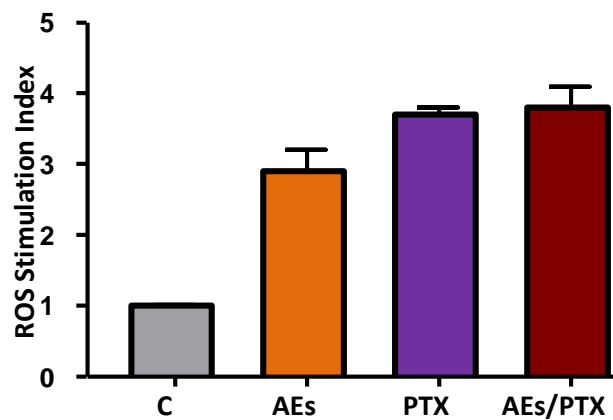

a

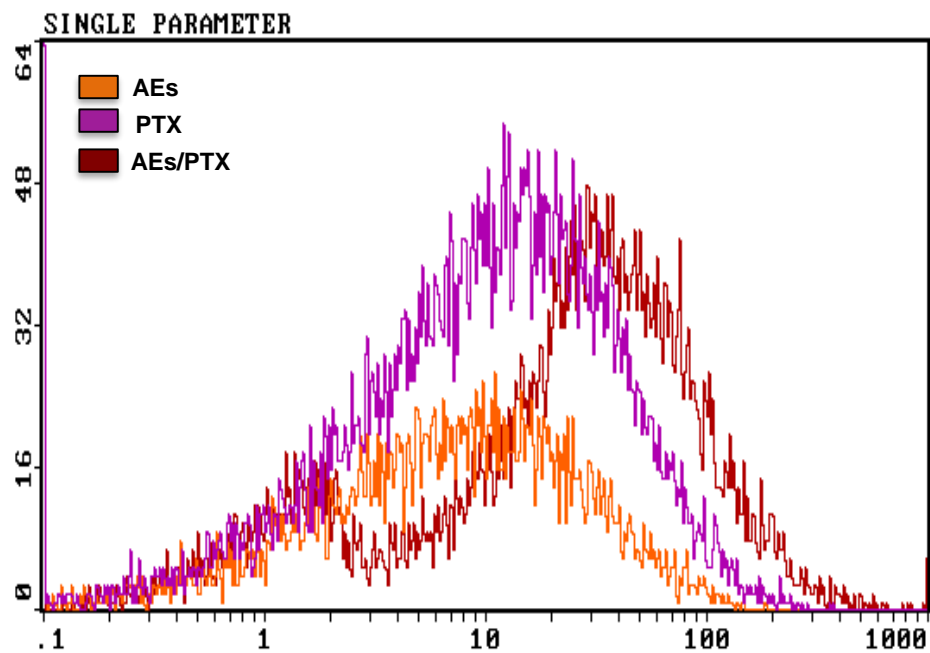

a1

Supplement: Supplementary Materials — integrate data described in the main document. Fig. 1s: effect of AEs on CDDP treated breast cancer cells. Cell viability assay: MDA-MB231 (a) and MCF7 (b) were treated with CDDP (20 μM) with or without AEs (from 12.5 to 50 μM) for 24 h. Histograms show cell viability. No significant differences are detected between combined treatment vs. CDDP alone. Fig. 2s: effect of AEs/PTX on regulated cell death. Apoptosis and autophagy marker analysis: cells were incubated with AEs or AEs/PTX and then assayed via western blot for c-PARP (a) and LC3 (b). Fig. 3s: cloning efficiency. Colony-forming analysis: MCF7 cells were exposed to AEs or AEs/PTX for 24 h and then allowed to grow and form colonies for subsequent 14 d. Cell colonies, after staining with crystal violet (a), were counted and the values reported as percent colony number in the histogram (b). C vs. PTX ∗∗p = 0.0049, 12.5 μM AEs vs. 12.5 μM AEs/PTX ∗∗∗p < 0.0001, and 25 μM AEs vs. 25 μM AEs/PTX ∗∗∗p = 0.0005. There is no significant difference between colony numbers of cells treated with AEs/PTX or PTX alone. Fig. 4s: ROS production in MDA-MB231 cells after 24 h of treatment. (a, a1) Flow cytometry: the mean fluorescence intensity was expressed as stimulation index obtained by ratio between ROS levels released by cells after 24 h of treatment and ROS detection in control cells. Data is the mean ± SD of 3 independent experiments. Indicative fluorescence peaks of ROS production in cells after 24 h of treatment with 25 μM AEs (red graph), 20 nM PTX (purple graph), and AEs/PTX, respectively, 25 μM and 20 nM (brown graph) are reported in (a1). There is no significant difference between groups. Fig. 5s: graphical abstract. Schematic representation of two convergent signaling pathways involved in synergistic effect of AE/PTX combined treatment. The figure suggests the potential role of artichoke polyphenols as sensitizers of chemotherapy in breast cancer cells. [file 7965435.f1.zip › Fig 4s.pdf]

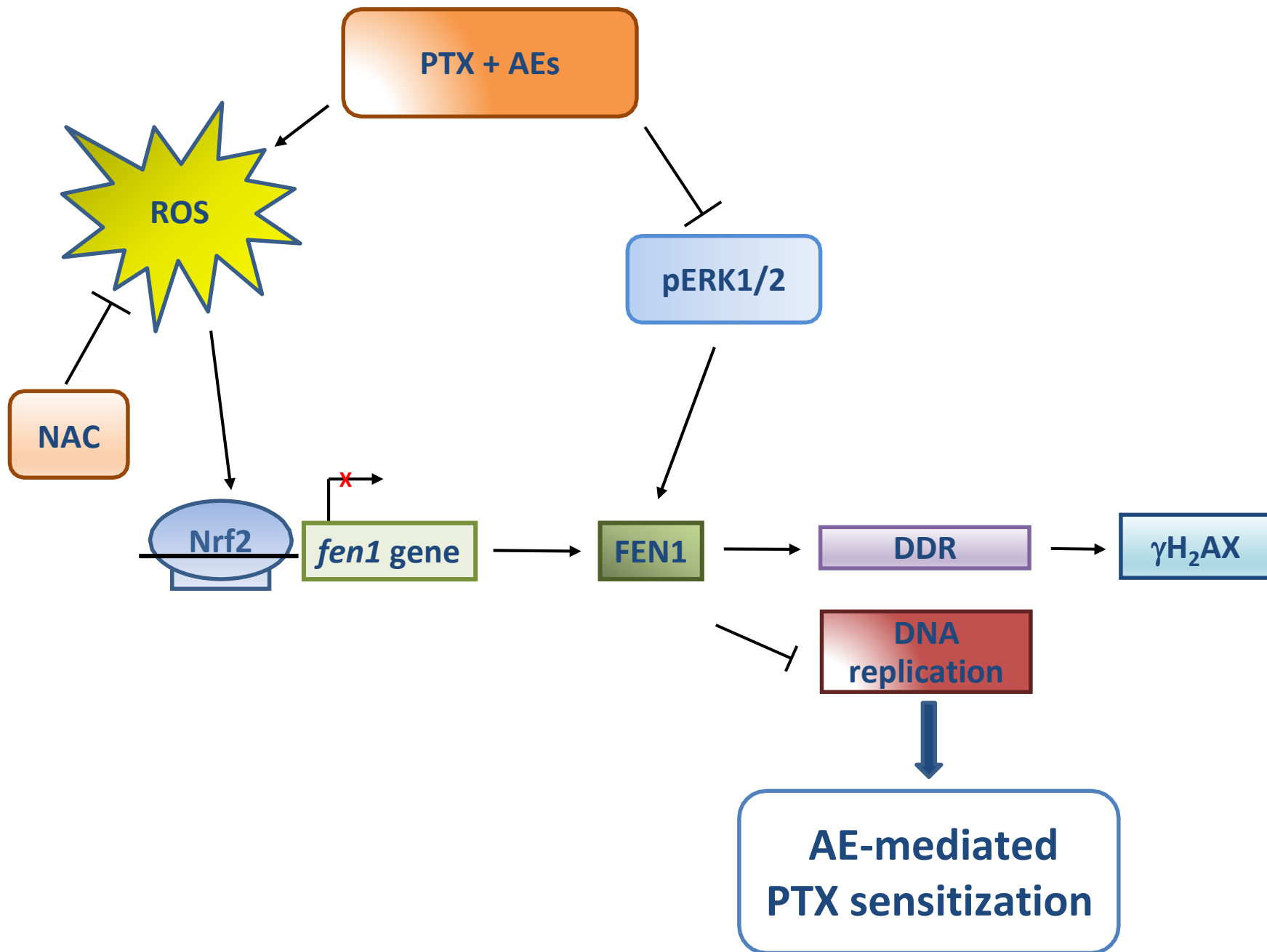

Fig.5s

Supplement: Supplementary Materials — integrate data described in the main document. Fig. 1s: effect of AEs on CDDP treated breast cancer cells. Cell viability assay: MDA-MB231 (a) and MCF7 (b) were treated with CDDP (20 μM) with or without AEs (from 12.5 to 50 μM) for 24 h. Histograms show cell viability. No significant differences are detected between combined treatment vs. CDDP alone. Fig. 2s: effect of AEs/PTX on regulated cell death. Apoptosis and autophagy marker analysis: cells were incubated with AEs or AEs/PTX and then assayed via western blot for c-PARP (a) and LC3 (b). Fig. 3s: cloning efficiency. Colony-forming analysis: MCF7 cells were exposed to AEs or AEs/PTX for 24 h and then allowed to grow and form colonies for subsequent 14 d. Cell colonies, after staining with crystal violet (a), were counted and the values reported as percent colony number in the histogram (b). C vs. PTX ∗∗p = 0.0049, 12.5 μM AEs vs. 12.5 μM AEs/PTX ∗∗∗p < 0.0001, and 25 μM AEs vs. 25 μM AEs/PTX ∗∗∗p = 0.0005. There is no significant difference between colony numbers of cells treated with AEs/PTX or PTX alone. Fig. 4s: ROS production in MDA-MB231 cells after 24 h of treatment. (a, a1) Flow cytometry: the mean fluorescence intensity was expressed as stimulation index obtained by ratio between ROS levels released by cells after 24 h of treatment and ROS detection in control cells. Data is the mean ± SD of 3 independent experiments. Indicative fluorescence peaks of ROS production in cells after 24 h of treatment with 25 μM AEs (red graph), 20 nM PTX (purple graph), and AEs/PTX, respectively, 25 μM and 20 nM (brown graph) are reported in (a1). There is no significant difference between groups. Fig. 5s: graphical abstract. Schematic representation of two convergent signaling pathways involved in synergistic effect of AE/PTX combined treatment. The figure suggests the potential role of artichoke polyphenols as sensitizers of chemotherapy in breast cancer cells. [file 7965435.f1.zip › Fig. 5s.pdf]
